# Environmental correlates of geographic divergence in a phenotypic trait: A case study using bat echolocation

**DOI:** 10.1002/ece3.3251

**Published:** 2017-08-09

**Authors:** Tinyiko Maluleke, David S. Jacobs, Henning Winker

**Affiliations:** ^1^ Department of Biological Sciences Animal Evolution and Systematics Group (AES) University of Cape Town Cape Town South Africa; ^2^ Centre for Statistics in Ecology Environmental and Conservation (SEEC) South African National Biodiversity Institute Cape Town South Africa

**Keywords:** acoustic signals, climate, horseshoe bats, resting frequency, selection

## Abstract

Divergence in phenotypic traits may arise from the interaction of different evolutionary forces, including different kinds of selection (e.g., ecological), genetic drift, and phenotypic plasticity. Sensory systems play an important role in survival and reproduction, and divergent selection on such systems may result in lineage diversification. Such diversification could be largely influenced by selection in different environments as a result of isolation by environment (IbE). We investigated this process using geographic variation in the resting echolocation frequency of the horseshoe bat species, *Rhinolophus damarensis*, as a test case. Bats were sampled along a latitudinal gradient ranging from 16°S to 32°S in the arid western half of southern Africa. We measured body size and peak resting frequencies (RF) from handheld individual bats. Three hypotheses for the divergence in RF were tested: (1) James’ Rule, (2) IbE, and (3) genetic drift through isolation by distance (IbD) to isolate the effects of body size, local climatic conditions, and geographic distance, respectively, on the resting frequency of *R. damarensis*. Our results did not support genetic drift because there was no correlation between RF variation and geographic distance. Our results also did not support James' Rule because there was no significant relationship between (1) geographic distances and RF, (2) body size and RF, or (3) body size and climatic variables. Instead, we found support for IbE in the form of a correlation between RF and both region and annual mean temperature, suggesting that RF variation may be the result of environmental discontinuities. The environmental discontinuities coincided with previously reported genetic divergence. Climatic gradients in conjunction with environmental discontinuities could lead to local adaptation in sensory signals and directed dispersal such that gene flow is restricted, allowing lineages to diverge. However, our study cannot exclude the role of processes like phenotypic plasticity in phenotypic variation.

## 1 INTRODUCTION

Every region has its own geological past, with unique flora and fauna shaped by environmental variation and barriers to gene flow that exist between geographic areas (Neuweiler, 2000). This is reflected in variation not only between species but also within species distributed over different environmental regions. Phenotypic divergence among populations of the same species may be the result of spatial isolation and drift (isolation by distance, IbD; Wright, 1943) in combination with selection for environment‐specific traits (isolation by environment, IbE a subset of isolation by adaptation; Safran et al., 2016). Both isolation and selection are likely to vary spatially along a continuum of environmental gradients (e.g., distance and climate). However, traits that are heritable and have a profound impact on fitness (e.g., sensory traits) are more likely to be impacted by natural selection than drift, resulting in adaptation to novel environments (Boughman, 2002; Campbell et al., 2010; Endler, 1992). Nevertheless, the role of phenotypic plasticity in phenotypic variation cannot be excluded (Ghalambor, McKay, Carroll, & Reznick, 2007; Thibert‐Plante & Hendry, 2011; Via et al., 1995). Phenotypic plasticity can influence variation if it promotes successful dispersal and survival in different environments (Pfennig et al., 2010). Such dispersal and resultant gene flow may favor the evolution of increased phenotypic plasticity, over adaptive genetic divergence, because it would promote adaptation to new environments within a few generations (Pfennig et al., 2010), thereby promoting phenotypic variation.

Sensory signals (e.g., acoustic signals) are important in the context of lineage diversification (Mutumi, Jacobs, & Winker, 2016; Odendaal, Jacobs, & Bishop, 2014; Slabberkoorn & Smith, 2002) because they are essential to the survival and reproduction of animals that rely on such signals for orientation and foraging as well as in mate choice and assortative mating (Bolnick & Kirkpatrick, 2012; Coyne & Orr, 2004). These signals are propagated through the environment, and specific environmental conditions can influence the evolutionary trajectory of the signaling system. Acoustic signals, in particular, are part of a sensory system that relies on audition and the propagation of sound through the atmosphere.

Atmospheric conditions (i.e., climate) can therefore exert a strong influence on geographic variation of complex signals, such as bird song (Lengagne & Slater, 2002), frog mating calls (Lingnau & Bastos, 2007), and the echolocation calls of bats (Lawrence & Simmons, 1982; Luo, Kosel, Zsebok, Siemers, & Goerlitz, 2014). These acoustic signals may diverge along climatic gradients as a result of variation in atmospheric attenuation of sound. Atmospheric attenuation, caused by the scattering and absorption of sound by the atmosphere, is the result of a complex interaction between the humidity and temperature of the air as well as the frequency of the sound (Hartley, 1989; Lawrence & Simmons, 1982; Luo et al., 2014; Mutumi et al., 2016). Climate could therefore play a pivotal role in driving the evolution of signaling systems through its effect on atmospheric sound attenuation (Griffin, 1971; Richards & Wiley, 1980;). For example, climatic conditions were found to have influenced the echolocation call frequency of *Hiposideros ruber* (Guillén, Juste, & Ibáñez, 2000) and some horseshoe bat species (Mutumi et al., 2016) through atmospheric attenuation of acoustic signals. Furthermore, other studies have suggested that bat echolocation call frequency diverges along climatic gradients (Snell‐Rood, 2012).

Such environmentally mediated differences in sensory systems leading to lineage diversification can be facilitated by geographic or environmental isolation of populations (Schluter, 2001). Isolation by geographic barriers and distance can influence trait variation by restricting gene flow between populations. This is usually manifested by an association between genetic or phenotypic differences and geographic distances and referred to as isolation by distance (IbD; Wright, 1943). Alternatively, when a species is distributed across several biomes, discontinuities in habitat can also result in environmentally mediated ecological isolation of local populations and a restriction of gene flow among them. Gene flow can be restricted in such situations either by selection against dispersers moving between populations in different habitats or by individual preference for remaining in a particular habitat (Nosil, 2004; Nosil, Vines, & Funk, 2005). This is usually manifested by an association between phenotypic differences and environmental differences (IbE, Crispo, Bentzen, Reznick, Kinnison, & Hendry, 2006; Nosil, 2012; Rundle & Nosil, 2005; Shafer & Wolf, 2013).

Bat echolocation has evolved primarily for orientation (Schnitzler et al., 2003; Schuchmann & Siemers, 2010; Simmons & Stein, 1980) and food acquisition (Heller & Von Helversen, 1989). There is ample evidence that the frequency of bat echolocation is adapted to the habitat and foraging mode of bats (Aldridge & Rautenbach, 1987; Jones & Holderied, 2007). There is also some, but not conclusive evidence, that it is secondarily involved in communication (Bastian & Jacobs, 2015; Knörnschild, Jung, Nagy, Metz, & Kalko, 2012; Siemers, Beedholm, Dietz, Dietz, & Ivanova, 2005). Echolocation thus provides an opportunity to investigate the role of evolutionary processes in the geographic variation of a phenotypic trait that is essential to survival and reproduction. However, only a few studies have investigated geographic variation in resting frequency and the environmental factors (humidity and temperature) responsible for it (Luo et al., 2014; Mutumi et al., 2016).

A potentially confounding factor in understanding the effect of climate on geographic variation of resting frequency is the inverse correlation between body size and echolocation frequency in bats (Jones, 1996, 1999). Larger bats tend to have lower frequencies, because they have longer and thicker vocal chords as well as larger resonant chambers (Jacobs, Barclay, & Walker, 2007). James' Rule (JR) proposes that animals occurring in hot humid environments generally have smaller body sizes than animals of the same species that occur in cooler, dryer environments, and the largest animals are expected to occur in cool, dry areas (James, 1970). This is because selection at cooler environments would favor larger individuals with lower surface area‐to‐volume ratio, and large body size enables individuals to conserve heat in cooler environments (Meiri & Dayan, 2003). Any variation in echolocation frequency might therefore simply be the result of the response of body size to different temperatures and humidity.

The southern Africa horseshoe bat, *Rhinolophus damarensis* Roberts, 1946, has a wide distribution in southern Africa which stretches from the more arid regions in northwestern South Africa and southern Namibia to the more mesic regions of northern Namibia and southern Angola (Jacobs et al., 2013). Genetic analyses based on mitochondrial cytochrome b and the nuclear thyrotropin beta chain precursor indicated that this species consists of two lineages, a northern lineage restricted to the more mesic regions of northern Namibia and a southern lineage restricted to the more arid regions of central and northwestern South Africa, extending into central Namibia. The phylogenetic analyses on cyt b revealed that the two lineages split ~4.8 MYA. There was a 4.1% sequence divergence between the northern and southern lineages with much lower within lineage divergence of 0.7% and 0.5%, respectively (Jacobs et al., 2013). *Rhinolophus damarensis* therefore provides an ideal opportunity for testing the roles of spatial separation and environment on the diversification of an acoustic signal. If IbE plays a dominant role in echolocation frequency divergence then: (1) call frequency variation should be correlated with climatic variables across populations; (2) call frequency variation should be partitioned in accordance with regional groupings; that is, there should be a strong signal of IBE between the northern and southern lineages; and (3) if call frequency is the result of selection rather than lineage‐specific differences (e.g., arising by genetic drift), the correlation between call frequency and climate should also be evident within a particular region and in the same direction as the correlation across all populations. Furthermore, call frequency variation should not be correlated with geographic distance; that is, there should be no signal for IbD. Under James' Rule, we predicted that there should be a correlation between body size and climatic factors and between body size and call frequency. We currently lack data on gene flow and dispersal to adequately test the role of phenotypic plasticity and other evolutionary processes, but we discuss their potential influence in promoting phenotypic variation.

## 2 METHODS

### 2.1 Study animal

*Rhinolophus damarenesis* (Jacobs et al., 2013) is a small insectivorous bat with a relatively high echolocation frequency (85.4 ± 1.4 kHz) and a mean forearm length of 49.5 ± 1.7 mm (Jacobs et al., 2013). It has a wide distribution in the western half of southern Africa stretching from western South Africa through Namibia to southwest Angola (Jacobs et al., 2013). This region is characterized by a wide range of arid and mesic climatic conditions.

**Figure 1:**
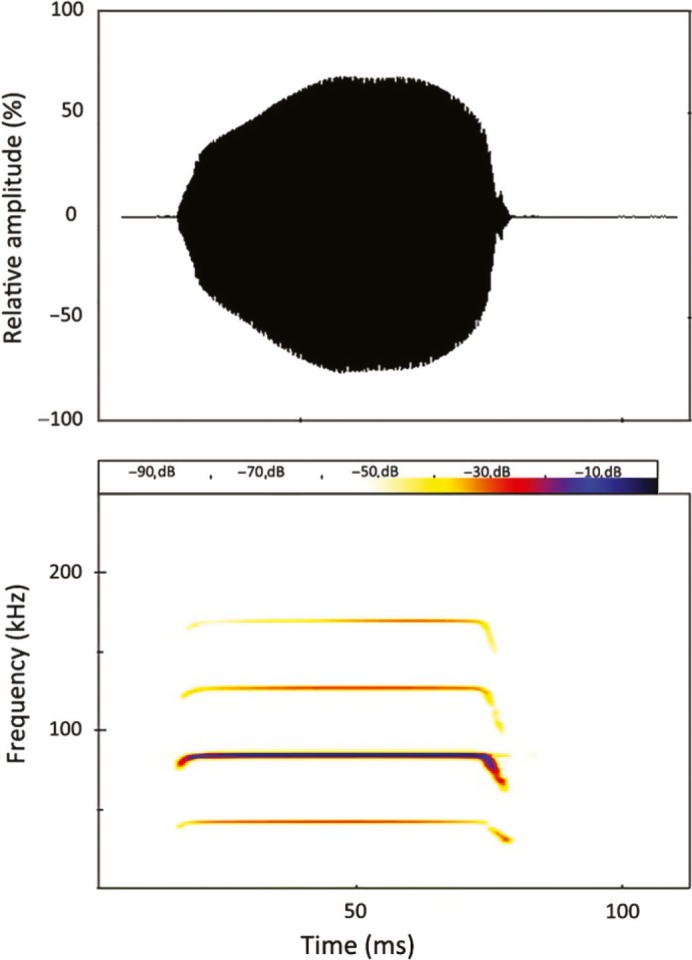
A typical call of *Rhinolophus damarensis*. Top = oscillogram; Bottom = spectrogram

Like other horseshoe bats (*Rhinolophidae*), *R. damarensis* uses high duty cycle (HDC) echolocation. Duty cycle is the ratio of call duration to call period and is usually expressed as a percentage. Rhinolophids have several harmonics in their echolocation calls but invariable concentrate call energy in the second harmonic (Fenton, 1994; Hartley & Suthers, 1988; Figure 1). Their calls (Figure 1) have a prominent constant frequency (CF) component preceded and followed by a frequency modulated (FM) component (Neuweiler, 1984). Horseshoe bats are perch hunters that are able to echolocate from a resting position while scanning the environment for prey (Jacobs et al., 2007). The peak frequency (frequency of maximum amplitude) of the CF component of a call emitted while at rest is called the resting frequency (RF). This RF is 100–300 Hz lower than the reference frequency, a narrow range of frequencies to which the acoustic fovea (a region of over‐representation of neurons in the auditory cortex) is tuned (Schuller & Pollak, 1979). Resting frequencies, which are the calls used when foraging from a perch, can therefore be recorded from handheld bats. This eliminates variance in frequency caused by horseshoe bats compensating for Doppler shifts in the returning echo during flight (Schnitzler & Denzinger, 2011).

### 2.2 Ethics statement

Methods used in this research were carried out in accordance with guidelines of the American Society of Mammalogists (Gannon & Sikes, 2007). We followed the sampling techniques/guidelines outlined by Kunz and Parsons (2009), and these were approved by the Science Faculty Animal Ethics Committee of the University of Cape Town (clearance number 2013/2012v6/DJ). All research was conducted under permits from the permitting authorities in the respective countries (Namibia—1429/2010; South Africa—AAA003‐00030‐0035; 1197/2008). Lands accessed to sample bats were publicly or privately owned and sometimes protected. At all times, the necessary permission was obtained. We did not sample protected or endangered species.

### 2.3 Sampling

**Figure 2:**
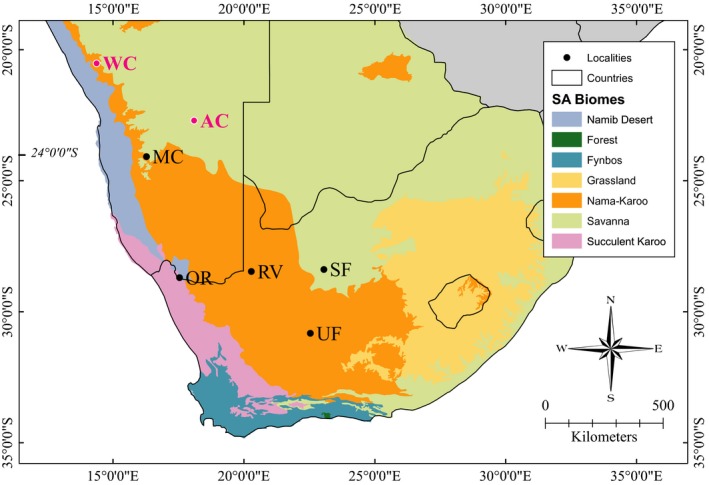
Major biomes of southern Africa (Rutherford, 1997) and the sampling localities for *Rhinolophus damarensis*. Key to abbreviations: Wondergat Cave (WC), Arnhem Cave (AC), Märcker Cave (MC), Orange River (OR), Riemvasmaak (RM), Soetfontein (SF), and Untjiesburg Farm (UF). The populations in red comprise the northern lineage, and those in black comprise the southern lineage (see Jacobs et al., 2013)

Data were collected across the known range of *R. damarensis*, in southern Africa from seven localities along a latitudinal gradient ranging from 16°S to 32°S (Figure 2). The geographic coordinates (latitude and longitude) of each locality at which we sampled *R. damarensis* were recorded using a Garmin GPS (model Colorado, Garmin International Inc, Kansas). Bats were captured from their roosting areas such as caves, mines, abandoned buildings, old mine shafts, hollow trees, and culverts under roads using hand nets during the day, or mist nets and harp traps as bats emerged from the roosts at dusk. Mist nets and harp traps were checked regularly throughout the trapping period to ensure that bats were not trapped in these for too long. All captured bats were transferred directly into soft cotton holding bags.

### 2.4 Morphology

The body mass of each bat was measured using a portable electronic balance (to the nearest 0.01 g), and forearm length (FA) was measured to the nearest 0.01 mm using digital calipers. Juveniles were excluded from this study and were identified by the presence of epiphyseal plates in their finger bones. The plates were detected by transilluminating the bats' wings (Anthony, 1988). Forearm length instead of mass was used as an indicator of body size because mass varies diurnally, seasonally and with life‐history stage (Jacobs et al., 2013; Mutumi et al., 2016). The sex of each bat was also recorded.

### 2.5 Echolocation

Echolocation calls were recorded from handheld bats positioned 10 cm in front of an Avisoft Ultrasound Gate 416 (Avisoft Bioacoustics, Berlin, Germany) microphone connected to a HP Compaq nx7010 notebook computer running Avisoft SasLab Pro software (Avisoft Bioacoustics Schönfließer, Germany) with the sampling rate set at 500 kHz. We measured 10 high‐quality calls (high signal‐to‐noise ratio) that occurred after the first 10 calls in each recording because horseshoe bats tune into their peak resting frequency after a period of silence (Schuller & Suga, 1976; Siemers et al., 2005). Calls were analyzed in BatSound Pro (version 3.20; Pettersson Elektronik AB, Uppsala, Sweden) using a sampling rate of 44,100 Hz (16 bits, mono) and a threshold of 15. We measured the peak resting frequency (kHz) from the fast Fourier transformation (FFT) power spectrum (size set at 1,024 samples) of the dominant 2nd harmonic in each call for 10 high‐quality (high signal‐to‐noise ratio) calls. The average RF over the 10 calls for each bat was used in the analyses.

### 2.6 Environmental variables

ArcGIS shape files for climate data were downloaded from BIOCLIM (http://www.worldclim.org/bioclim) and OEI (www.en.openei.org) websites. The shape files were analyzed in ArcGIS v.10 to extract data on relative humidity (RH) and annual mean temperature (AMT). Relative humidity was based on data taken at 10 m above the surface of the earth by NASA Surface meteorology and solar energy (SSE Release 6.0, Data Set, November 2007), a 22‐year (1983–2005) monthly and annual average dataset (http://eosweb.larc.nasa.gov/sse/). Annual mean temperature shape files were based on monthly temperature values that were averaged over the period of 50 years (1950–2000). All environmental data were extracted for a radius of 10 km around each locality at which we sampled *R. damarensis*. A radius of 10 km was used because the home range of rhinolophids of similar size to *R. damarensis* (e.g., *R. euryale* and *R. ferremequinum*) has been measured at within 10 km (Bontadina, Schofield, & Naef‐Daenzer, 2002; Flanders & Jones, 2009; Goiti, Aihartza, Garin, & Zabala, 2003; Jones & Morton, 1992). Currently, there are no data on the home range of *R. damarensis*.

### 2.7 Atmospheric attenuation and detection range

We calculated the mean atmospheric attenuation for each locality and then used these to calculate the mean detection ranges of prey (PDR) for each population of *R. damarensis* using the web calculator developed and described in Stilz and Schnitzler (2012). Prey detection ranges were calculated to determine the impacts of ecological constraints on the echolocation performance of *R. damarensis* (Denzinger & Schnitzler, 2004; Fenton et al., 1995; Neuweiler, 1990) at each locality. The calculations required the following information: (1) climatic conditions (e.g., AMT, RH, and atmospheric pressure) at each sampled site, where atmospheric pressure was kept at that for normal atmospheric conditions, taken as 101.325 pascal; (2) resting frequency (Hz) of each individual bat; (3) the dynamic range, which is the difference between peak intensity (dB SPL) measured at 1 m and the auditory threshold of the bat (assumed to be 0 dB SPL for horseshoe bats (Holderied & von Helversen, 2003; Long & Schnitzler, 1975); (4) reflection loss, C_1_, which accounts for the fraction of the energy reflected, and (5) geometric spreading, C_2_, which quantifies the loss of energy due to spreading multiplied by 2 for both outgoing emitted call and the returning echo. The values of the latter two factors are dependent on the geometry of the reflected wave. The web calculator generates C_1_ and C_2_ depending on the target selected. We chose the point function reflector which resembles the insect prey of bats. We used a call intensity of 123.7 dB SPL for (c) above, measured by Fawcett, Jacobs, Surlykke, and Ratcliffe (2015) for a horseshoe bat of similar size (*R. capensis*). The actual call intensity of *R. damarensis* is currently unknown. We compared the calculated PDRs for the different localities using the Kruskal–Wallis test followed by multiple comparisons.

### 2.8 Predictor variables

To investigate the plausibility of the James Rule, IbE, and IbD, we evaluated candidate models with different combinations of environmental, spatial, and biological predictor variables to determine their influence on RF across the distributional range of *R. damarensis*.

Biological predictor variables included forearm length (FA), which was used as a proxy for body size (James' Rules) and gender which was used to determine whether there was sexual dimorphism in RF within *R. damarensis*.

The four alternative environmental variables considered were annual mean temperature (AMT), relative humidity (RH), atmospheric attenuation (AA), and prey detection range (PDR). Temperature and relative humidity have previously been suggested as factors largely responsible for atmospheric attenuation of sound (Hartley, 1989; Lawrence & Simmons, 1982; Luo et al., 2014). Atmospheric attenuation and PDR represent ecological variables that combine the effects of temperature and relative humidity. To avoid collinearity between these environmental variables, we fitted them separately in alternative candidate models. Because RF is used in the calculation of both AA and PDR (REF), we standardized the values of AA and PDR by the mean RF calculated across populations. This avoided statistical circularity between response (RF) and predictor variables (AA and PDR).

Similarly, the predictors region (Reg) and latitude (Lat) were considered as alternative covariates in separate candidate models. The factor Reg comprised two levels representing two genetically differentiated lineages within *R. damarensis* (Jacobs et al., 2013). One lineage included populations that were located north of latitude −24ºS and was designated the northern lineage. The other lineage included populations further south than latitude −24ºS and was designated the southern lineage (Figure 2). By contrast, the covariate Lat represented a linear predictor, which would imply a continuous geographic divergence in RF instead of a geographic break. The split between the two *R. damarensis* lineages was considered to provide insight into potential spatial divergence of RF in *R. damarensis*.

### 2.9 Statistical procedure

Geographic variation in RF may be the result of stochastic processes (e.g., genetic drift). One way in which such stochastic processes would be manifested is in a positive relationship between the RF of populations and the geographic distance between them. We therefore also determined whether differences in RF were associated with geographic distances by calculating a dissimilarity matrix of RF (kHz) differences among localities and Euclidean distances among populations from their geographic coordinates (longitude and latitude). A simple pairwise Mantel test was employed to analyze associations between RF differences and geographic distances (Legendre & Fortin, 2010). We used 10,000 permutations based on Monte Carlo simulation tests.

We applied linear mixed‐effects models (LMEs) to analyze the relationship between the response variable (RF) and the environmental (AMT or RH or AA or PDR), spatial (Lat or Reg) and the biological (FA, sex) predictor variables. Linear mixed‐effects models incorporate both fixed and random effects (Verbeke & Lesaffre, 1996; Zuur, Ieno, Walker, Saveliev, & Smith, 2009), where the fixed effects quantify the overall effects across the different localities and the random effects quantify variation of the fixed effect parameters across localities (Bolker et al., 2008). The random effect for sampling location was included to account for the nested sampling design as a result of sampling several individuals from a single location. In addition, LMEs can incorporate autocorrelation in the data, which was considered here to account for potential spatial dependencies among sampled localities (Bjørnstad & Falck, 2001) to ensure that IbE is not being driven by spatial autocorrelation (Shafer & Wolf, 2013). It was also for this reason that we investigated IbD (Shafer & Wolf, 2013).

We further explored whether selection or stochastic processes were responsible for RF variation by running separate LMEs on just the southern populations. If RF variation is the result of selection under different climates, the correlation between climatic variables and RF among all populations should also exist and be in the same direction among the southern populations.

**Figure 3:**
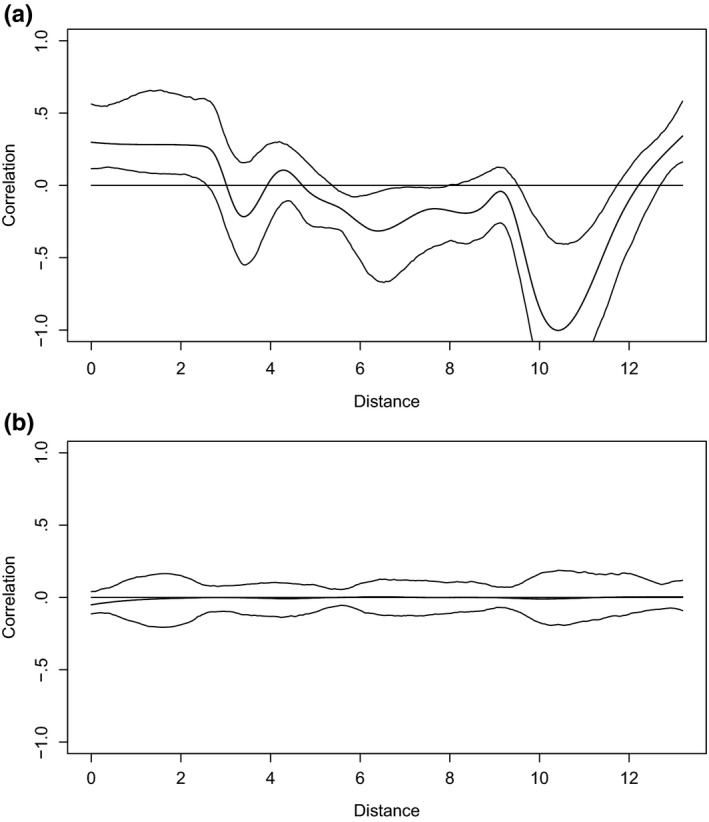
Spatial autocorrelation due to strong spatial dependencies in observed data (a) and (b) a graph with no spatial autocorrelation after spatial dependencies had been accounted for by the simple random‐effects model

**Figure 4:**
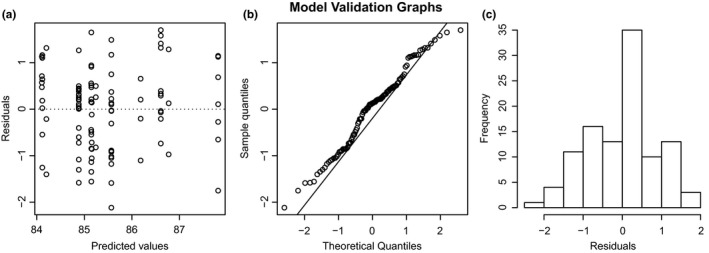
Model validation graphs showing residuals closely approximating a normal distribution with no violation of the assumption of homogenous variance. The graphs are (a) predicted values against residuals that are clearly spread out, (b) a linear relationship between sample quantiles and theoretical quantiles, and (c) histogram showing a normal distribution between frequency and residuals

We first used a reasonably complex set of variables (AMT, Reg, and Sex with resting frequency as the response variable) to determine the best mixed‐effects structure (Zuur et al., 2009). Llinear mixed‐effects models were fitted with and without spatial correlation structures and a random effect for sampling locality. The candidate LMEs were fitted with various spatial autocorrelation functions on longitude and latitude coordinates, including corGaus, corExp, corRatio, corSpher, and AR1. However, the most parsimonious error structure, as judged by the Akaike's information criterion (AIC), was a linear mixed model (LME) that only included a random effect for locality. This was also supported by nonparametric spatial spline correlograms based on the model residuals, which showed that spatial autocorrelation, evident for a fixed‐effects linear model, could be effectively accounted for by including the random effect for locality (Figure 3). Inspection of residuals from the random‐effects LME showed that the residuals closely approximated a normal distribution and provided no evidence for violation of the assumption of homogenous variance (Figure 4). The LME with a random effect for locality was then selected as the most parsimonious error model for analyzing the variation in RF.

To determine the optimal combination of covariates, competing models were ranked based on their AICc, which is the AIC corrected for a small sample size (*n*) relative to the number of parameters being estimated (*K*). We ran a total of 40 model permutations to examine which model subsets best explained the variation in our data. Akaike information criterion corrected differences (Δ_*i*_) and Akaike weights (*w*_*i*_) were used to identify the best candidate models (Amar, Koeslag, Malan, Brown, & Wreford, 2014; Odendaal et al., 2014). Models with the lowest AICc and highest Akaike values were considered the most parsimonious, and the differences in AIC scores (Δ_*i*_) were calculated to determine the likelihood that a given model was the best approximating model relative to other candidate models (Amar et al., 2014; Odendaal et al., 2014; Symonds & Moussalli, 2011). A model with a (Δ_*i*_) value of zero was considered the best approximating model, and those with values of up to two were regarded as good as the best model (Symonds & Moussalli, 2011). Evidence ratios of the best fitting models were calculated relative to the other subsequent candidate models to determine the relative evidence for the best approximating model in relation to the other candidate models (Amar et al., 2014).

The predictions of James' Rule were tested by incorporating forearm in our models because body size can influence resting frequency variation in echolocating bats due to the allometric relationship between body size (e.g., forearm) and resting frequency (Jacobs et al., 2007; Jones, 1996, 1999).

All our analyses were conducted in R version 3.1.2, and the following packages were used: “AICcmodavg” for model selection and multimodel inference to compare and rank multiple competing models and to estimate those that best approximate the true processes underlying the biological phenomenon under study (Symonds & Moussalli, 2011); “lme” for fitting the linear mixed‐effects model and to incorporate both fixed and random effects in a linear predictor expression from which the conditional mean of the response was evaluated (Bates, Mächler, Bolker, & Walker, 2015); “effects” for displaying the effect sizes of linear, generalized linear, and other models (Fox & Andersen, 2006); “car” a companion to applied regression for regression diagnostics and other regression‐related tasks (Fox, 2002); “ncf” a spatial nonparametric covariance function to make correlograms and to check for spatial autocorrelation (Bjørnstad & Falck, 2001); and “Ade4” for estimating geographic distances between sites (Legendre & Fortin, 2010; Thioulouse, Chessel, Dole′Dec, & Olivier, 1997).

## 3 RESULTS

We recorded and analyzed calls from 106 adult *R. damarensis* individuals from seven localities (Table 1). This species had an average RF of 85.43 ± 1.3 kHz and average call duration of 31.14 ± 5.9 ms. Mean RF ranged from 84.4 ± 0.7 kHz to 87.6 ± 1.1 kHz across localities. Females had mean RFs ranging from 84.5 ± 0.4 kHz to 87.8 ± 1.0 kHz, while the RF of males ranged from 84.4 kHz ± 0.9 to 86.9 ± 1.1 kHz.

**Table 1:** The forearm length, resting echolocation frequency (mean ± *SD*), environmental variables and calculated atmospheric attenuation and prey detection range for each locality at which *Rhinolophus damarensis* was sampled. The number of individuals per population is shown in parentheses. Localities are shown in the order of increasing latitude from north to south. The northern populations are designated by a “N” next to their names. Sample sizes are given in parentheses

	Locality						
	Wondergat^N^	Arnhem^N^	Märcker	Soetfontein	Riemvasmaak	Orange River	Uintjiesberg
FA (mm) Mean ± *SD*	50.6 ± 1.5	51.8 ± 1.9	50.5 ± 1.9	48.9 ± 2.8	49.8 ± 1.2	49.1 ± 1.6	52.3 ± 1.1
Range	47.1–51.82	46.8–54.8	48.1–52.7	43.3–51.4	47.6–51.1	45.5–51.6	50.4–54.6
	(15)	(17)	(6)	(7)	(10)	(29)	(22)
RF (kHz) Mean ± *SD*	84.4 ± 0.7	85.0 ± 0.9	84.7 ± 1.1	85.7 ± 0.8	87.6 ± 1.1	85.9 ± 1.3	84.9 ± 0.7
Range	82.9–85.3	83.8–86.1	82.8–85.7	84.7–86.5	85.8–88.9	83.5–88.3	83.3–86.2
	(15)	(17)	(6)	(7)	(10)	(29)	(22)
F‐RF (kHz) Mean ± *SD*	84.5 ± 0.4	85.1 ± 1.0	85.2 ± 0.7	86.1 ± 0.7	87.9 ± 1.0	87.1 ± 0.8	84.9 ± 0.7
Range	84.1–85.3	83.6–86.1	84.4–85.7	85.1–86.8	86.1–89.0	85.9–88.3	83.3–86.1
	(7)	(13)	(3)	(4)	(7)	(11)	(22)
M‐RF (kHz) Mean ± *SD*	84.4 ± 0.9	84.8 ± 0.3	84.1 ± 1.4	85.2 ± 0.5	86.9 ± 1.1	85.2 ± 1.0	–
Range	82.9–85.2	84.6–85.2	82.8–85.5	85.3–85.7	85.8–88.1	83.4–87.1	–
	(8)	(4)	(3)	(3)	(3)	(18)	–
Lat	−20.51	−22.70	−24.08	−28.38	−28.70	−28.70	−30.83
Reg	North	North	South	South	South	South	South
RH (%)	38.25	38.47	32.80	41.75	35.9	39.15	41.54
AMT (°C)	21.67	19.19	15.78	18.32	20.24	18.82	15.53
AA (dB/m)	2.56 ± 0.02	2.32 ± 0.02	1.84 ± 0.03	2.36 ± 0.02	2.41 ± 0.03	2.33 ± 0.04	2.05 ± 0.02
PDR (m)	8.87 ± 0.06	9.54 ± 0.07	11.27 ± 0.14	9.43 ± 0.08	9.25 ± 0.11	9.49 ± 0.12	10.42 ± 0.06

AA, atmospheric attenuation; AMT, annual mean temperature; FA, forearm length; F‐RF, female resting frequency; Lat, latitude; M‐RF, male resting frequency; PDR, prey detection range; Reg, region is divided into the northern and southern regions; RF, resting frequency; RH, relative humidity. Number of individuals (*n*) per population is shown in parentheses.

Differences in RF were not associated with geographic distances. A simple pairwise Mantel test revealed that there was no significant association between resting frequency differences and geographic distances in *R. damarensis* (Monte Carlo test observation: 0.084, 10,000 replicates, *p *=* *.5474).

The LME model selection suggested that the three most supported models all included AMT as an environmental predictor (Table 2), which together resulted in an accumulative weight (Cum.wt) of 77%. In the order of highest ranking based on Akaike information criterion weight (highest AICwt), these three models were Reg + AMT + Sex, Reg + AMT + Sex + FA, and Lat + AMT + Sex. However, the evidence ratio indicated that the first model (Reg + AMT + Sex) was 3 and 9 times stronger than the second and third models, respectively (Table 2). Each of the three variables in the most parsimonious model had a *p*‐value of <.05 (Table 3).

**Table 2:** Results from linear mixed‐effects models (LMEs) testing for association between the resting frequency of *Rhinolophus damarensis* and environmental variables

Model	K	AICc	Delta_AICc	ModelLik	AICcWt	LL	Cum.wt	ER
**Reg + AMT + Sex**	**6**	**295.89**	**0.000**	**1.000**	**0.535**	**−141.52**	0.535	
Reg + AMT + Sex + FA	7	298.11	2.218	0.330	0.176	**−**141.48	0.712	3.03
Lat + AMT + Sex	6	300.37	4.476	0.107	0.057	**−**143.76	0.769	8.93
Reg + PDR + Sex	6	300.85	4.956	0.084	0.045	**−**144.10	0.814	11.91
Reg + AA + Sex	6	301.16	5.271	0.072	0.038	**−**144.16	0.852	13.95
Sex	4	302.58	6.690	0.035	0.019	**−**147.09	0.879	28.1
Lat + AMT + Sex + FA	7	302.66	6.769	0.034	0.018	**−**143.76	0.889	28.37
Reg + PDR + Sex + FA	7	303.13	7.236	0.027	0.014	**−**143.99	0.903	37.26
Reg + AA + Sex + FA	7	303.44	7.545	0.023	0.012	**−**144.15	0.916	43.49
AMT + Sex	5	303.79	7.897	0.019	0.010	**−**146.6	0.926	51.87
Lat + PDR + Sex	6	304.08	8.186	0.017	0.009	**−**145.62	0.935	58.04
Lat + AA + Sex	6	304.09	8.195	0.017	0.009	**−**145.62	0.944	58.15
PDR + Sex	5	304.14	8.245	0.016	0.009	**−**146.77	0.952	61.7
AA + Sex	5	304.34	8.443	0.015	0.008	−146.87	0.960	68.13
Lat + RH + Sex	6	304.43	8.539	0.014	0.007	−145.79	0.968	69.02
RH + Sex	5	304.63	8.739	0.013	0.007	−147.02	0.975	79
Reg + RH + Sex	6	305.29	9.392	0.009	0.005	−146.22	0.979	>100
AMT + Sex + FA	6	306.04	10.144	0.006	0.003	−146.59	0.983	>100
Lat + PDR + Sex + FA	7	306.37	10.480	0.005	0.003	−145.62	0.986	>100
Lat + AA + Sex + FA	7	306.38	10.489	0.005	0.003	−145.62	0.988	>100
PDR + Sex + FA	6	306.39	10.491	0.005	0.003	−146.77	0.991	>100
AA + Sex + FA	6	306.58	10.690	0.005	0.003	−146.87	0.994	>100
Lat + RH + Sex + FA	7	306.72	10.827	0.004	0.002	−145.79	0.996	>100
RH + Sex + FA	6	306.88	10.984	0.004	0.002	−147.01	0.998	>100
Reg + RH + Sex + FA	7	307.58	11.686	0.003	0.002	−146.22	1.000	>100
Reg + AMT	5	318.52	22.623	0.000	0.000	−153.96	1.000	>100
Lat + AMT	5	319.52	23.623	0.000	0.000	−154.46	1.000	>100
FA	4	320.32	24.426	0.000	0.000	−155.96	1.000	>100
Reg + PDR	5	321.04	25.144	0.000	0.000	−155.22	1.000	>100
Reg + AA	5	321.62	25.723	0.000	0.000	−155.51	1.000	>100
Lat	4	322.61	26.714	0.000	0.000	−157.11	1.000	>100
Reg	4	322.95	27.061	0.000	0.000	−157.28	1.000	>100
Lat + PDR	5	323.16	27.264	0.000	0.000	−156.28	1.000	>100
Lat + AA	5	323.23	27.340	0.000	0.000	−156.32	1.000	>100
Lat + RH	5	323.59	27.692	0.000	0.000	−156.49	1.000	>100
AMT	4	324.09	28.193	0.000	0.000	−157.85	1.000	>100
PDR	4	324.21	28.313	0.000	0.000	−157.91	1.000	>100
AA	4	324.42	28.523	0.000	0.000	−158.01	1.000	>100
RH	4	324.64	28.746	0.000	0.000	−158.12	1.000	>100
Reg + RH	5	325.08	29.186	0.000	0.000	**−**157.24	1.000	>100

AICc, Akaike information criterion scores; ΔAICc, change in AICc relative to the highest ranked model; AICwt, Akaike information criterion weight; AMT, Annual mean temperature; Cum.wt, cumulative weight; ER, evidence ratio; FA, forearm; K, number of parameters; Lat, latitude; LL, log‐likelihood; Reg, region; RH, relative humidity. Values for the strongest model are given in bold font at the top of the table.

**Table 3:** Summary statistics for the most parsimonious linear mixed‐effects model (LMEs) fitted by REML on the RF of *Rhinolophus damarensis*

Variable	Value	SE	DF	*t*‐value	*p*‐value
Intercept	74.42854	2.904708	98	25.62342	.0000
AMT	0.47396	0.140041	4	3.384406	.0179
Reg	2.26557	0.630106	4	3.59554	.0228
Sex	1.03812	0.201173	98	5.160326	.0000

AMT, annual mean temperature; DF, degree of freedom; FA, forearm; Reg, region; SE, Standard error.

All LMEs models indicated that forearm (FA) as a proxy for body size did not strongly influence resting frequency (RF) variation in *R. damarensis*. This was further supported by regression analysis which showed no correlation between FA and RF (*R* ^2^ = 0.02, *p* = .21).

**Figure 5:**
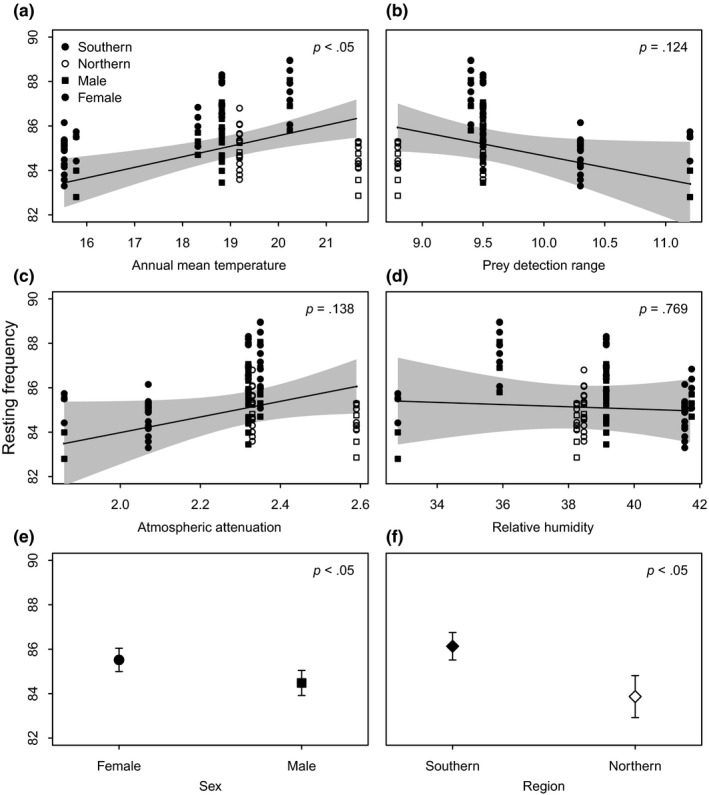
Predicted effects of (a) annual mean temperature (AMT); (b) prey detection ranges (PDR); (c) atmospheric attenuation (AA); (d) relative humidity (RH); (e) sexual dimorphism and (f) region on RF variation in *Rhinolophus damarensis*. The gray‐shaded areas and error bars represent 95% confidence intervals

RF of *R. damarensis* varied with AMT, exhibiting a positive correlation between RF and AMT (Figure 5a). Comparison of spatial (LAT and REG) and environmental (RH and AMT) factors using linear mixed‐effects models revealed that AMT and REG had the lowest AIC value. This, and the fact that LAT was not a significant predictor of RF, indicated that environmental discontinuities rather than continuous geographic divergence in RF strongly influenced resting frequency variation in *R. damarensis*. Prey detection range, AA, and RH were also not significant predictors of RF variation (Figure 5b–d). There was also sexual dimorphism in the RF of *R. damarensis*, and females had higher RF than males (Figure 5e).

The inclusion of Reg in the model indicated that the northern and southern regional groups, based on genetic lineages uncovered by Jacobs et al., 2013; had distinct RFs; the southern region had higher RF than the northern region (Figure 5f). However, the RFs of the two regions overlapped; the southern region had RFs which ranged from 82 to 89 kHz and that of the northern region ranged from 82 to 86 kHz.

Our separate linear mixed‐effects models on just the southern populations revealed that the model with the highest ranking was RF ~ AMT + Sex. None of the other variables (PDR, AA and RH) in the lower ranked models had a *p*‐value of <.05. Similar to the analyses which included all populations (Table 3), the association between AMT and RF was also positive (slope = 0.62279, *p* < .002).

The mean detection range and atmospheric attenuation of echolocation signals across populations of *R. damarensis* were 9.68 ± 0.63 m and 2.29 ± 0.19 dB/m, respectively. Across populations from the northern lineage, the mean atmospheric attenuation (2.43 ± 0.12 dB/m) was higher and mean detection range (9.23 ± 0.36 m) lower than across populations from the southern lineage (2.22 ± 0.18 dB/m and 9.87 ± 0.62, respectively). However, the populations at Märcker Cave and Uintjiesberg, which are the northernmost and southernmost populations, respectively, of the southern genetic lineage, had the lowest AAs and highest PDRs and differed from all other populations (Tables 1 and 4, Figure 2). Estimated PDRs between the two populations (Wondergat and Arhnem Cave) in the northern regions were significantly different but also differed from some, but not all, of the PDRs in the populations comprising the southern region (Kruskal–Wallis test: *H*(6, *N* = 106) = 91.8, *p *<* *.001; Table 4). These results suggest that the significance of region as a predictor for RF variation is also influenced by other environmental variables besides temperature and relative humidity and their effect on PDR.

**Table 4:** Results of multiple comparison tests of prey detection ranges among populations of *Rhinolophus damarensis*. Localities are listed in the order of increasing southern latitude. *p* Values are given in parentheses below the *z′* values. Bold font indicates significant differences

Locality	Wondergat^N^	Arnhem^N^	Märcker	Soetfontein	Riemvasmaak	Orange river	Uintjiesberg
Wondergat	–	**4.6**	**6.4**	2.3	1.2	**4.3**	**7.9**
		(**<.001**)	**(<.0001)**	(.4)	(1.0)	**(<.001)**	**(<.01)**
Arnhem	**4.6**	–	**3.1**	1.2	2.8	0.9	**3.1**
	**(<.001)**		**(<.001)**	(1.0)	(.1)	(1.0)	**(<.05)**
Märcker	**6.4**	**3.1**	–	**3.7**	**5.0**	**3.9**	1.0
	**(<.00001)**	**(<.05)**		**(<.01)**	**(<.001)**	**(<.01)**	(1.0)
Soetfontein	2.3	1.2	**3.7**	–	1.1	0.7	**3.7**
	(.4)	(1.0)	**(<.05)**		(1.0)	(1.0)	**(<.01)**
Riemvasmaak	1.2	2.8	**5.0**	1.1	–	2.4	**5.6**
	(1.0)	(.1)	**(<.0001)**	(1.0)		(.4)	**(<.00001)**
Orange River	**4.3**	0.8	**3.9**	0.7	2.4	–	**4.5**
	**(<.001)**	(1.0)	**(<.01)**	(1.0)	(.4)		**(<.001)**
Uintjiesberg	**7.9**	**3.2**	1.0	**3.7**	**5.6**	**4.5**	–
	**(<.00001)**	**(<.01)**	(1.0)	**(<.01)**	**(<.00001)**	**(<.001)**	

## 4 DISCUSSION

We found no evidence for James' Rule or for random genetic drift. Body size was not correlated with RF nor climatic variables, suggesting that variation in RF was not the result of concomitant variation in body size as proposed by James' Rule. Similarly, the Mantel test showed no IbD effect and there was therefore no evidence that genetic drift was responsible for the variation in RFs. In contrast, the LMEs indicated that there was support for IbE in the form of an association between RF and region, which was based on two geographically separated genetic lineages. Furthermore, RF variation was also associated with the climatic variable, AMT across all populations and a similar association was found across the southern populations. This suggests that regional differences in RF were not simply due to lineage‐specific differences but that local adaptation had exerted an influence. However, the relationship we found between RF and AMT is correlative rather than causative. Alternative evolutionary processes, for example, phenotypic plasticity, cannot therefore be excluded at this stage.

Most studies on geographic variation have focused on humidity as the main predictor of such variation (for a review see Jiang, Wu, & Feng, 2015) providing evidence that echolocating bats that are found in humid areas will experience severe atmospheric attenuation resulting in lower RF than those that are found in dry areas (Guillén et al., 2000; Heller & Von Helversen, 1989; Lawrence & Simmons, 1982). For example, geographic variation in the RF of *R. pusillus* was positively correlated with mean annual rainfall (Jiang et al., 2010). However, it is known that the propagation of echolocation calls and acoustic signals, in general, are influenced by both temperature and relative humidity (Lawrence & Simmons, 1982; Luo et al., 2014) through their effect on atmospheric attenuation. Atmospheric attenuation is the result of a complex interaction between temperature, humidity, and the frequency of the sound being propagated (Luo et al., 2014). For example, in two species of horseshoe bats, *R. simulator* and *R. swinnyi,* distributed in the more mesic eastern half of southern Africa, acoustic divergence in RF was influenced by the interaction between temperature and humidity and the degree of influence was higher in *R. swinnyi* than in *R. simulator* because *R. swinnyi* echolocated at higher frequencies (*R. swinnyi* = 103.77 ± 1.70 kHz; *R. simulator* = 80.32 ± 2.20 kHz; Mutumi et al., 2016). However, our results for *R. damarensis* indicate that temperature (AMT) was the predominant climatic factor responsible for the divergence in echolocation RF. This was also the case for *R. ferrumequinum* (Jiang et al., 2015) in Asia and for *R. capensis* in southern Africa. Similar to *R. damarensis*,* R. capensis* has a distribution that extends into the more arid western and northwestern regions of South Africa (Neumann & Bamford, 2015; Odendaal et al., 2014) and its RF was also not correlated with RH (Odendaal et al., 2014). This may be because both *R. damarensis* (Figure 5b) and *R. capensis* (Odendaal et al., 2014) occupy more arid regions (Table 1; compare with Mutumi et al., 2016) and in such areas temperature is a better predictor of RF than RH.

The effect of temperature is mediated by its interaction with RH (see Luo et al., 2014; Mutumi et al., 2016), and this interaction may vary from one region to another such that one or the other may exert the predominant influence on atmospheric attenuation and the propagation of acoustic signals. Our results and those of Mutumi et al. (2016) suggest that in warm mesic regions, both temperature and relative humidity are likely to influence the propagation of acoustic signals, but in warm arid regions, the predominant climatic factor that defines atmospheric attenuation is more likely to be temperature. The complex nonlinear interaction between temperature and humidity may also explain the paradoxical results obtained by studies on the climatic influence on geographic variation in acoustic signals. For example, geographic variation in RF was positively correlated with mean annual rainfall in *R. pusillus* (Jiang et al., 2010) but negatively correlated with mean annual rainfall in *Hipposideros ruber* (Guillén et al., 2000). These contrary findings are often attributed to the complexity of natural selection (e.g., Jiang et al., 2015) but may also be a consequence of the nonlinear effects and potential collinearity of temperature and humidity on atmospheric attenuation (Luo et al., 2014). Neither Jiang et al. (2010) nor Guillén et al. (2000) considered the interactive effects of temperature and humidity on atmospheric attenuation.

Divergence in acoustic signals may arise from the action of different evolutionary forces, among which environmental selection is the most common (Podos & Warren, 2007; Wilkins, Seddon, & Safran, 2013). In the case of *R. damarensis*, divergence in RFs may be the result of selection for optimal detection ranges within the respective habitats of the populations in the two regions. Populations of *R. damarensis* in the northern region have RF on the lower end of the observed range for populations in the southern region. The lower RF of populations from the northern region could be the result of the more mesic conditions in northern Namibia. Acoustic divergence within species can arise when signals undergo selection for optimal propagation of acoustic signals in the local acoustic environment (Wiley & Richards, 1982) leading to populations occupying different ecological niches (Nosil, 2012). Although we found significant differences in estimated PDRs between populations, there was no clear distinction between the PDRs of the two regions (Tables 1 and 4), suggesting that the ecological niches of the bats occupying these two regions, if different, may be defined by more than just atmospheric attenuation and its effect on PDR.

Nevertheless, the two regions have different climates. The northern part of Namibia is a more mesic region and characterized by woodland vegetation (Hoetzel, Dupont, & Wefer, 2015; Simmons, Griffin, Griffin, Marais, & Kolberg, 1998; White, 1983), while central Namibia and the Karoo ecoregions (Succulent and Nama) of South Africa are semi‐arid (Neumann & Bamford, 2015; Okitsu, 2005; Thuiller et al., 2006; Figure 2). The importance of region as a predictor of RF variation may be indicative of these environmental discontinuities.

If so, the coincidence of phenotypic difference with environmental discontinuities strongly suggests that divergence in this species is the result of IbE possibly facilitated by adaptation of sensory signals to local environmental conditions. Such environmental discontinuities in the context of the propagation of acoustic signals may be partly defined by atmospheric conditions such as temperature and humidity (this study; Jiang et al., 2015; Mutumi et al., 2016; ) but in all probability also by other unobserved environmental factors (i.e., latent variables). The association of latitude and altitude with RF variation in two species of horseshoe bats (Mutumi et al., 2016) and with region in *R. damarensis* suggests that in addition to temperature and humidity, other as‐yet‐unidentified environmental variables influence RF and this too may be suggestive of the influence of environmental discontinuities on phenotypic traits.

Geographic variation in RF between populations of *R. damarensis* in the two regions may have evolved as a consequence of adaptation to local environmental conditions. Such local adaptation could impede gene flow between the northern and southern regions occupied by *R. damarensis* (Gillam & McCracken, 2007; Rundle & Nosil, 2005) through several processes including selection against migrants (Hendry, 2004; Thibert‐Plante & Hendry, 2009) or matching habitat choice (Edelaar, Siepielski, & Clobert, 2008). The latter process is likely if bats, especially at the margins of suitable habitat (e.g., Märcker and Uintjiesberg populations in our study), choose habitats on the basis of maximizing their detection ranges. Such choice would result in a reduction in gene flow to habitats with decreased detection distances (e.g., the northern region) and possibly reduce gene flow between the two regions. A detailed study on the extent and direction of gene flow among *R. damarensis* populations and the incorporation of robust genetic data into the kinds of analyses conducted here are required to test this idea.

The genetic difference between the two lineages of *R. damarensi*s (Jacobs et al., 2013), although not conclusive, and the statistical support for differences in RF between the two regions is an indication that populations of *R. damarensis* may be in the process of diverging under selection. Several studies have revealed lineage diversification within species in the western half of southern Africa as a result of climate‐induced changes in biomes (Bauer & Lamb, 2005; Matthee & Flemming, 2002). Comparative studies in the western half of southern Africa revealed lineage diversification and clades within species in a wide range of animals such as reptiles (da Silva & Tolley, 2013), insects (Pitzalis & Bologna, 2010), and mammals (Matthee & Robinson, 1996; Willows‐Munro & Matthee, 2011; du Toit, Jansen Van Vuuren, Matthee, & Matthee, 2012) including horseshoe bats (Jacobs et al., 2013; Odendaal et al., 2014; Taylor et al., 2012). It appears therefore that local adaptation to climate in combination with an interruption of gene flow resulting from IbE may explain phenotypic divergence in *R. damarensis* and the regions fauna. Although this is supported by the similar relationship between AMT and RF reported here for all populations as well as for the southern group of populations considered separately, we do not know whether this relationship exists among the northern populations. Unfortunately, there were not enough populations in the northern region to repeat this analysis on the northern populations separately. There is therefore a possibility that the relationship between AMT and RF in the northern populations may be absent or reversed and that the variation, in at least the northern populations, may be the result of drift, phenotypic plasticity or some other process not considered here. We cannot therefore exclude the influence of such processes on *R. damarensis* with a high degree of confidence. Phenotypic plasticity, for example, can be advantageous if it results in the expression of traits that increase an individual's fitness in a new environment. In this way, the costs incurred from dispersal into different environments to the natal one can be minimized (Fitzpatrick, 2012; Pfennig et al., 2010). One potential limitation to plasticity is the tight coupling between RF and the acoustic fovea in high duty cycle bats, like *R. damarensis*, that use Doppler shift compensation (Neuweiler, 1984; Schnitzler & Denzinger, 2011). However, HDC bats are able to shift their RFs (up to 3.9 kHz) in response to both neighboring conspecifics and different ambient noise conditions (Hage, Jiang, Berquist, Feng, & Metzner, 2013; Hiryu et al., 2006). Although small, such shifts in frequency encompass the differences between the mean RFs of neighboring populations of *R. damarensis* and may allow bats to optimize their detection range under the local climatic conditions without a change in allele frequency. However, an adequate test of phenotypic plasticity requires greater knowledge of the genetic basis and heritability of phenotypic traits, of gene flow between populations and of interhabitat differences in foraging behavior of these bats.

Sexual dimorphism within a species may suggest that social rather than ecological selection is responsible for any divergence. Sex in *R. damarensis* was a significant predictor of RF with females generally echolocating at slightly higher RF than males (Figure. 5d). This is also true for several other horseshoe bat species (Chen, Jones, & Rossiter, 2009; Siemers et al., 2005; Yoshino et al., 2006). Bats exhibit a wide diversity of mating systems, but little is known about the mechanisms involved in courtship and mating (Grilliot, Burnett, & Mendonc, 2009). Recent evidence suggests that echolocation calls may be a source of information for individual recognition (Siemers & Kerth, 2006; Siemers et al., 2005). Sexual dimorphism in RF may have an essential social function by signaling the sex of the caller (Kazial & Masters, 2004), promoting mate recognition (Guillén et al., 2000) and reproductive success (Grilliot et al., 2009). It has been shown for some species that call frequencies of males and females do not overlap, and frequency may encode the sex of the signaler reliably (Neuweiler et al., 1987). However, this was not the case for *R. damarensis*. Instead, there was much overlap in male and female call frequency (Table 1), suggesting that social selection was not responsible. Similarly, it is unlikely that cultural drift played a role. Resting frequency variation may be the result of call frequency being passed from mother to infant through learning. In combination with female philopatry such learning could result in RF diverging among populations randomly by a process of cultural drift (see Jiang et al., 2015 for a review). However, the absence of any IbD signal and the association of RF variation with temperature and region suggest that other evolutionary processes rather than drift are responsible.

Recent studies suggest that climate change may have a severe negative impact on the sensory ecology of sound‐mediated behaviors (Luo et al., 2014). An increase in temperature and/or relative humidity, combined with the high RF used by *R. damerensis*, is likely to result in a decrease in the detection volume of *R. damarensis* under a global warming scenario (Luo et al., 2014). Global warming may therefore directly impact the sensory ecology of organisms reliant on sound‐mediated behaviors (Luo et al., 2014). If changing ambient temperature increases atmospheric attenuation, then echolocating species of bats may have reduced prey detection distances (Luo et al., 2014). Reduction in prey detection distances could lead to ineffective foraging and an increase in the costs–benefits ratio of foraging, which could lead to local extinction of populations.

Climate‐induced variation in acoustic signals may not be restricted to bats. There is also some support for climate‐induced geographic variation in bird song (Snell‐Rood, 2012), suggesting that lineage diversification in general may be driven by habitat‐mediated differences in acoustic signals, especially when such differences are accompanied by genetic differentiation. Climatic gradients in conjunction with other ecological discontinuities could lead to local adaptation and/or phenotypic plasticity with directed dispersal (Edelaar et al., 2008) such that gene flow is restricted, allowing lineages to diverge.

## AUTHOR CONTRIBUTIONS

TM and DSJ conceived and designed the experiments. TM and DSJ performed the experiments. TM, HW, and DSJ analyzed the data. DSJ and HW contributed reagents/materials/analysis tools. TM, DSJ, and HW wrote the manuscript.

## CONFLICT OF INTEREST

None declared.
